# Tailored therapy for severe asthma

**DOI:** 10.1186/2049-6958-10-1

**Published:** 2015-01-16

**Authors:** Francesco Menzella, Mirco Lusuardi, Carla Galeone, Luigi Zucchi

**Affiliations:** Department of Cardiac-Thoracic-Vascular and Intensive Care Medicine, Pneumology Unit, IRCCS- Arcispedale Santa Maria Nuova, Viale Risorgimento 56, 42123 Reggio Emilia, Italy; Unit of Respiratory Rehabilitation, AUSL Reggio Emilia, S. Sebastiano Hospital, Correggio, Italy

**Keywords:** Asthma, Cytokines, COPD, Inflammation, Monoclonal antibodies

## Abstract

Patients with severe asthma or COPD have often a suboptimal symptom control due to inadequate treatment. A better understanding of pathogenetic mechanisms, phenotypes, endotypes and the new technologies available in the fields of molecular biology and immunogenetics have made it possible to synthesize specific monoclonal antibodies virtually able to interact with any target antigen, or to open a way for new therapeutic target options. At the moment, the only biologic drug available in clinical practice is omalizumab. To overcome the limits of omalizumab, the research has focused on new monoclonal antibodies presenting higher avidity for IgE (e.g. ligelizumab and lumiximab) and ability to interact also with low affinity IgE receptor (FcϵRII). At present, many new biological drugs with different mechanisms of action and targets are matter of research. It is very important to identify the asthmatic phenotype in order to select the most appropriate drug for the individual patient. The most promising agents are targeted against cytokines of Th2 pattern and related receptors, such as IL-2 (daclizumab) and IL-13 (lebrikizumab) or IL-5 in patients with hypereosinophilia (mepolizumab, reslizumab and benralizumab). Other interesting drugs have as a target TNF-α or its soluble receptor (infliximab, golimumab and etanercept) or IL-1 (canakinumab), a cytokine with an important systemic proinflammatory action. Finally, the discovery of increased levels of C5a in the airways of asthmatic patients has led to the synthesis of a specific monoclonal antibody (eculizumab). Further help should come from the identification of biomarkers that can guide in choosing the best treatment for the individual patient, such as IgE for omalizumab or periostin for lebrikizumab.

## Introduction

Patients with severe asthma have often a suboptimal symptom control due to inadequate therapeutic options. Actually, there is an increasing need to identify new molecules effective to overcome treatment limitations, particularly through the remarkable implementation of the research in the pathophysiology and immunology fields.

The earliest and most important pathophysiological mechanism of asthma is represented by airways inflammation, predisposing to exacerbations and probably to bronchial remodelling [1]. It is well known that asthma is a complex disorder with many different phenotypes whose definition is based on clinical, inflammatory or causative factors [2]; and heterogeneous inflammatory profiles have been described, such as eosinophilic, neutrophilic and paucigranulocytic [3]. A better knowledge of the different phenotypes of asthma should drive the most appropriate treatment.

## Review

The discovery of different patterns of inflammation and the transition to the next level of complexity by molecular phenotyping and development of biomarkers [4, 5] have led to a further and significant step forward, thanks to new technologies in molecular biology and immunogenetics. These findings have made it possible to synthesize specific monoclonal antibodies [MoAb(s)] interacting with any target antigen and have opened the way for the development of tailored therapeutic options. omalizumab is the first and, at present, the only MoAb available in clinical respiratory medicine for the treatment of asthma. The biological drugs studied so far (Table 1) have also shown to be effective in other respiratory diseases or allergic reactions, such as Churg-Strauss syndrome, hypereosinophilic syndrome, eosinophilic pneumonia, nasal polyposis, or atopic dermatitis, with promising perspectives in the clinical setting.

**Table 1:** **Monoclonal antibodies and their targets**

Name	Target	Study phase	Route of administration
Omalizumab	IgE	Approved	Subcutaneous
Quilizumab	IgE	IIa	Subcutaneous
Ligelizumab	IgE	IIa	Subcutaneous
Lumiliximab	FcϵRII (CD23)	II/III	Oral
Daclizumab	IL2-R (CD25)	II	Intravenous
Lebrikizumab	IL-13	III	Subcutaneous
Mepolizumab	IL-5	III	Intravenous/Subcutaneous
Reslizumab	IL-5	III	Intravenous
Benralizumab	IL-5	IIb	Intravenous
Mogamulizumab	CCR4	III	Intravenous
Infliximab	TNF-α	II	Intravenous
Golimumab	TNF-α	IIa	Intravenous
Etanercept	TNF-α (soluble receptor)	II	Subcutaneous
Eculizumab	C5a	II	Intravenous
Canakimumab	IL-1ß	IIb	Subcutaneous
SNG001 (Inhaled IFN-β 1a)	IFN- β	II	Inhalation

### Blocking IgE. Omalizumab, but non only

**Figure 1:**
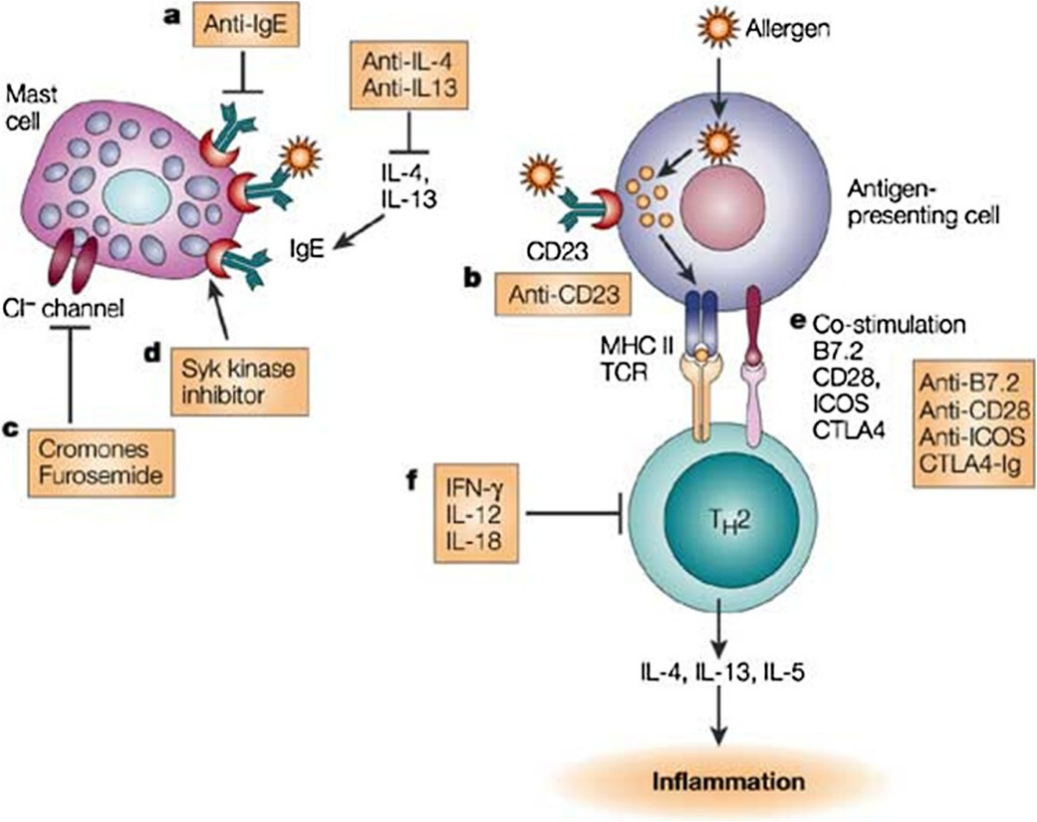
**Mechanism of action of omalizumab (Modified from** [9] **).**

Based on currently available data, the IgE are at the heart of the immuno-allergen-induced inflammation. Omalizumab (Xolair®) is a murine monoclonal antibody (MAE11) produced with the somatic cells hybridization method, whose main characteristic is a paratope that can bind to high (FcϵRI) and low affinity (FcϵRII) IgE receptors on the cell membrane of basophils and mast cells, inhibiting the degranulation and activation of cellular mediators (Figure 1). Several clinical trials have been recently performed in order to evaluate the clinical effectiveness of omalizumab in severe allergic uncontrolled asthma patients. These studies have shown its effectiveness and safety, with a significant reduction in the rate of asthma exacerbations (up to 50%), improvement of quality of life scores [6] and steroid-sparing effect [6]. Omalizumab dosage is based on total IgE levels combined with body weight [7]. At the moment, there are no validated biomarkers identifying potential responders among patients with asthma, with a promising exception represented by periostin according to some recent data [8].

The effectiveness of omalizumab has been recently demonstrated in non-allergic asthma patients on long-term treatment [10]. These data support the hypothesis of a local production of IgE without systemic sensitization [11].

Other authors confirmed the efficacy of omalizumab in children with severe asthma living in urban centers in the United States [12, 13] and in cases of allergic diseases such as urticaria, atopic dermatitis, allergy to Hymenoptera venom, oculorhinitis, sinusitis, allergic bronchopulmonary aspergillosis, and Churg-Strauss syndrome (CSS) [14]. Omalizumab has been used in CSS and uncontrolled asthma patients on high-dose steroid therapy, with excellent results in terms of asthma control and significant decrease in peripheral eosinophilia [15]. There were also some reports on CSS development related to the administration of anti-IgEMoAb, probably due to the reduction of systemic steroids rather than to omalizumab [16]. An interesting case report showed positive results with omalizumab in a patient with relapsing chronic eosinophilic pneumonia (CEP), asthma, allergic sensitization to mites and pollens and high levels of IgE, without recurrence of CEP after 15 months of anti-IgE treatment [17]. A flow cytometry analysis showed alterations in the expression of CD23 (FcϵRII) on B lymphocytes, which can correlate with the response to omalizumab, along with possible effects on down-regulation of FcϵRI and FcϵRII receptors [18].

According to current data, omalizumab is indicated as a regular long-term treatment: as a matter of fact, IgE levels and FcϵRI receptors increase after 3–4 weeks from discontinuation of the drug. However, a study on a small group of patients treated for 6 years showed a substantial clinical stability 3 years after stopping omalizumab [19]. Two recent Italian papers also report some increase in long-term clinical benefits [20, 21], with good cost-effective profile in saving healthcare resources [21, 22].

To overcome limitations of omalizumab, an expected progress has been the production of monoclonal antibodies with greater avidity for IgE, such as RG7449, a new humanized MoAb that binds to the M1 segment of membrane IgE and has as a target the B-lymphocytes before they are activated to produce IgE [23].

The QGE031B/ligelizumab is a new humanized antibody, highly potent inhibitor of the high affinity IgE receptor (FceRI). As determined by surface Plasmon resonance, QGE031B has a Kd = 130 pM for human IgE, representing an almost 50-fold improvement in affinity for human IgE as compared to omalizumab (Kd = 6.8 nM).

The effectiveness of QGE031B is being assessed in patients with allergic asthma, (4/5 clinical severity stage according to GINA guidelines), in a phase IIa clinical trial, with omalizumab as active comparator.

Lumiliximab is another promising agent, a humanized anti CD23 IgG1 antibody (IDEC-152) used successfully in recurrences of lymphocytic leukemia [24]. Due to its ability to reduce the concentration of circulating IgE through the activation of FcϵRII (CD23) it has been tested in patients with mild asthma, but without particularly significant clinical outcomes.

### Th2 lymphocytes and their citokyne pattern

It has long been known that asthma is a disease linked to the activation of helper Th2-lymphocytes (whose key cytokines are mainly IL-2, IL-3, IL-4, IL-5, IL-9, IL-13 and GM-CSF) with a concomitant lack of inhibition by T_reg_ lymphocytes for defective production of IL-10 [25, 26].

Soluble receptors can be a potential target for new drugs, being the immunoglobulin fusion proteins and recombinant RNA able to interfere with the Th2 cytokines or their receptors.

Allergen activation of Th2 cells stimulates IL-2 secretion and the expression of its receptor IL-2R. Daclizumab is a monoclonal antibody directed against CD25 subunit of IL2-R that inhibits different cellular functions, such as proliferation and cytokine production. The effects of this drug were evaluated in a randomized clinical trial where 115 severe asthma patients were randomized to intravenous daclizumab or placebo and the results showed only small improvements in lung function and asthma control [27].

Interleukin 13 (IL-13) promotes the production of IgE by plasma cells, the release of eosinophil chemoattractants and the contraction of airways smooth muscle cells.

A preliminary study on patients with moderate allergic asthma tested the use of two anti-IL-13 monoclonal antibodies, one blocking the specific epitope for IL4-R and the second binding the epitope that recognizes IL13-R. Positive results were found only for anti IL4-R activity in terms of reduced fall in FEV_1_ after allergen inhalation challenge [28].

The anti-IL-13 IgG4 monoclonal antibody lebrikizumab was compared to placebo in uncontrolled asthma patients. Lebrikizumab improved FEV_1_, particularly in the subgroup with high values of FeNO and periostin in peripheral blood [29]. Periostin is an indirect index of IL-13 activity and candidates as a promising biomarker to identify potential responders to the treatment. No differences emerged regarding exacerbations, symptom score and use of rescue medication.

### Eosinophils and IL-5

The maturation, recruitment and survival of eosinophils in the respiratory tract are determined by IL-3, GM-CSF, but especially by IL-5, which has a key role in mediating airways eosinophilic inflammation in asthmatic patients [30]. IL-5 induces the final differentiation of activated B cells into antibody-forming cells and enhances the proliferation and differentiation of eosinophil precursors into mature eosinophils [31]. In murine models it seems also involved in the airways remodeling, so that the administration of antibodies antagonizing IL-5 largely prevents the sub-epithelial and peribronchial fibrosis induced by the inhalation of allergens [32].

Different conditions are associated with eosinophilia, such as asthma, atopic diseases, helminth infestation, drug hypersensitivity and malignancies; for these reasons several monoclonal antibodies such as anti-IL5 mepolizumab (Bosatria®), reslizumab and benralizumab have been studied.

Mepolizumab is a humanized antibody that binds IgG1κ-type IL-5 preventing its linking to the specific receptor. A first study was conducted on the treatment of idiopathic hypereosinophilic syndrome [33] and Churg-Strauss syndrome, in which a significant reduction was demonstrated in the administration of systemic corticosteroids with a good disease control [34].

The available data show a reduced number of exacerbations in steroid-dependent asthma patients with a concomitant reduction of sputum eosinophils; on the contrary, lung function and bronchial hyperresponsiveness did not improve [35]. A more recent work suggested that mepolizumab administered at a dose of 75, 250 and 750 mg significantly reduces the need for systemic steroids and the number of exacerbations in severe eosinophilic asthma patients [36]. The efficacy of this drug in terms of improved asthma control and discontinuation of systemic steroids was evident only in trials focused on persistently hypereosinophilic patients, confirming the need for defining asthma phenotypes [37]. In a randomized, double-blind trial involving 135 patients with severe eosinophilic asthma requiring daily oral glucocorticoid therapy to maintain control, mepolizumab had a significant glucocorticoid-sparing effect, reduced exacerbations, and improved control of asthma symptoms [38]. Another recent, randomized, double-blind, double-dummy study, involved 576 patients assigned to receive mepolizumab administered as either a 75-mg intravenously dose or a 100-mg subcutaneously dose, or placebo every 4 weeks for 32 weeks. The rate of exacerbations was reduced by 47% among patients receiving intravenous mepolizumab and by 53% among those receiving subcutaneous mepolizumab, as compared to those receiving placebo [39].

A clinical trial tested reslizumab in poorly controlled asthma and airways eosinophilia. A significant reduction of sputum eosinophils was found with a parallel significant improvement in quality of life, FEV_1_, and disease control, in particular reference to a reduction of exacerbations [40].

Benralizumab is another drug tested in clinical trial. It is a monoclonal antibody directed against the alpha chain of IL-5 receptor, administered intravenously. In clinical trials benralizumab reduced the levels of peripheral eosinophils in moderate asthma patients, its effects persisting up to 8–12 weeks [41]. Other authors demonstrated that benralizumab induces antibody dependent cell-mediated cytotoxicity on eosinophils and basophils, resulting in a significant reduction of blood eosinophils and their bone marrow precursors [42].

There are several other trials testing new molecules, such as humanized antibody IgG1 anti-hIL-5 Ra (MEDI-563), antisense oligonucleotides, targeted monoclonal antibodies against chemokine receptor CCR3 [43].

Chemokine receptor 4 (CCR4) is a target of mogamulizumab (Poteligeo®), a defucosylated humanized monoclonal antibody. It was approved in Japan in March 2012 for the treatment of refractory T-cell leukemia, relapsing peripheral T cell lymphoma (PTCL) and cutaneous T-cell lymphoma [44]. Mogalizumab is currently licensed to Amgen for developments in non-oncological indications, and in 2011 a phase 1 study was initiated in adult asthmatic patients [44].

### The role of TNF-α

**Figure 2:**
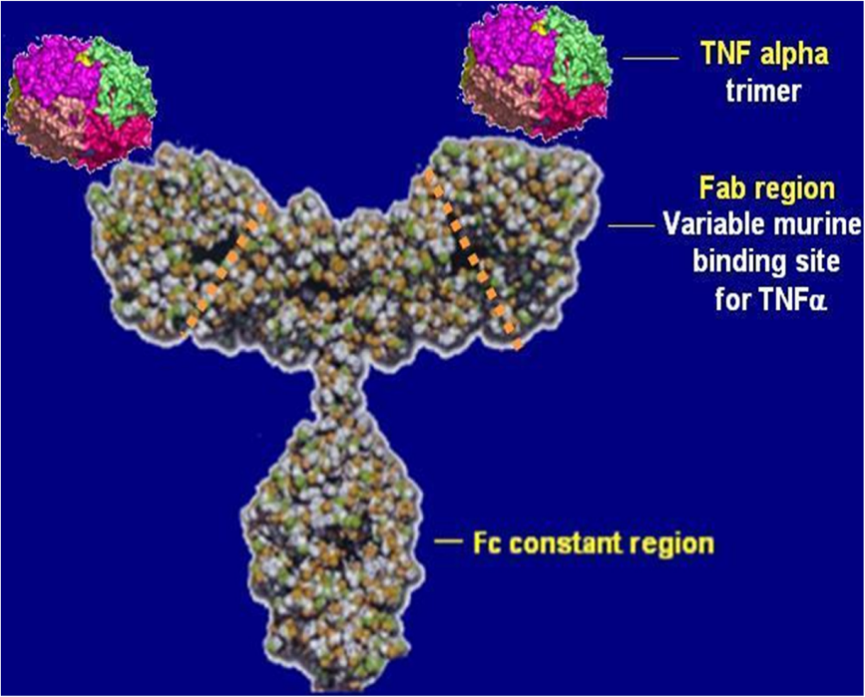
**Protein structure of infliximab (Modified from** [45] **).**

Tumor necrosis factor-alpha (TNF-α) is a cytokine with a variety of effects such as stimulation and inhibition of cell growth, angiogenesis, cytotoxicity, inflammation and immunomodulation. It is therefore involved in different inflammatory and respiratory diseases including asthma and COPD. The interest on TNF-α has developed in the last few years as a result of studies that showed an increase of this cytokine in bronchoalveolar lavage fluid of patients with severe asthma. The anti-TNF-α mainly include infliximab, etanercept, and golimumab; the first two are monoclonal antibodies that bind to and inactivate TNF-α (Figure 2), the third is a fusion protein between the p75 TNF receptor and the Fc domain of human IgG1 that can bind TNF-α with a half-life longer than the native receptor.

Among clinical studies on etanercept (Enbrel®) a few showed good results in reducing bronchial hyperreactivity [46], whereas others poor clinical efficacy in terms of lung function improvement and quality of life [47]. Other authors showed a small but significant increase in quality of life without differences on lung function [48]. Interesting data have emerged from a small series of 7 overweight women in whom infliximab improved asthma control in terms of reduction of oral steroids, exacerbations and hospitalizations. Overweight is a known condition of TNF-α overexpression [49].

A trial of infliximab (Remicade®) administered to patients with mild-to-moderate asthma showed no significant improvements in morning PEF, symptom score and use of rescue medication [50]. The results of a large multicenter trial of patients with severe asthma treated for 12 months with golimumab (Simponi®) versus placebo have not proved exciting, because of the failure to reduce flare-ups and the lack of improvement in FEV_1_, with an unfavourable risk-benefit profile [51].

TNF inhibitors have also been studied in COPD, although not extensively. A trial of infliximab administered to patients with mild to moderate COPD did not show benefits on symptom score, lung function and exacerbations; an improvement of 6-minute walk test (6MWT) in the group treated with infliximab was not statistically significant. However, the active treatment had to be discontinued for adverse events in a high percentage of cases (20-27%) as compared to controls (9%) [52].

An observational study conducted to evaluate the efficacy of TNF-α antagonists in preventing hospitalizations in patients with rheumatoid arthritis and COPD showed that treatment with etanercept was associated with a reduction of 50% in the rate of hospitalization [53].

It must be remembered that the use of TNF-alpha inhibitors is potentially burdened by infectious risks, such as reactivation of latent tuberculosis and histoplasmosis, congestive heart failure, lymphoproliferative and demyelinating disorders [54, 55], so their application in respiratory diseases is further limited. Nevertheless, the studies on TNF-alpha inhibitors should not be abandoned, given the significant and not yet fully understood heterogeneity of asthma that does not allow us, at the moment, to identify which phenotype could possibly benefit from these drugs.

### A new frontier in asthma treatment: the complement system

The complement system is composed of several plasma proteins playing a central role in the immune response, producing substances that are involved in the inflammatory response cascade, causing vasodilation and increased vascular permeability (C3a, C5a).

The plasma components of complement are present in the circulation in an inactive form; activation may occur through a “classical pathway” (i.e. formation of antigen-antibody complexes) or an “alternative pathway” (mediated e.g. by endotoxins, complex polysaccharides, or IgA). The key point is represented, in both cases, by the activation of the C3 fraction.

Recent studies have shown that in asthmatic airways there is an increase in C5a levels, and C5a receptor expression on bronchial epithelium and smooth muscle cells. Furthermore, in animal (mice) preclinical trials the inhibition of C5a cleavage improves lung function [56].

Eculizumab (Soliris®) is a new humanized IgG2/4 k monoclonal antibody produced with the recombinant DNA technology by the NS0 cell line. It binds to C5 preventing its cleavage by the C5 convertase and the following formation of C5a and C5b-9 complex.

Eculizumab was approved for the treatment of paroxysmal nocturnal hemoglobinuria. A placebo-controlled trial with this drug administered by inhalation for the inhibition of intrapulmonary C5 showed a significant response in mild allergic asthma patients, in terms of improved spirometry and reduction of late allergen-induced eosinophilia in induced sputum [56].

### Proinflammatory cytokines

Interleukin 1 (IL-1) is a cytokine secreted by macrophages, monocytes, fibroblasts and dendritic cells in response to microbial infections through stimulation by LPS (an endotoxin produced by gram negative bacteria), TNF and following interaction with CD4 lymphocytes [57]. It has pro-inflammatory effects such as fever, vasodilation and stimulation of other cytokines, e.g. IL-2 that activates Th2 lymphocytes. There are two types of IL-1, alpha and beta, with different molecular structure but similar function acting on the same receptor. The IL-1 receptor antagonist (IL-1Ra) with a structure similar to IL-1 is a natural inhibitor of these molecules.

In asthma, the response against aeroallergens is mainly based on the inflammatory response of T lymphocytes, with an important involvement of IL-1β in the inflammatory signal transduction. Canakinumab (Ilaris®) is a humanized monoclonal antibody developed for the treatment of a group of rare and potentially lethal autoinflammatory diseases, denominated cryopyrin-associated periodic syndromes (CAPS). These are represented in particular by the familial cold autoinflammatory syndrome (FCAS), characterized by overproduction of IL-1 beta [56]. Canakinumab can induce a long-lasting and selective blockade of IL-1 beta, blocking the inflammation cascade in several autoimmune disorders, and has been included in the list of “Orphan Drug” by the FDA (Food and Drug Administration) and EMA (European Medicines Agency) [58]. A randomized double-blind clinical trial evaluated the safety and tolerability of canakinumab in mild allergic asthma patients, determining its anti-inflammatory effects on late asthmatic response after allergen inhalation, with good results compared to pre-treatment phase [59]. Despite these encouraging data, at present there are no further studies in progress on the drug, at least in asthma.

### IFN-β and viral infections

Exacerbations of asthma caused by respiratory viruses are a large unmet medical need, especially in the most severe forms of the disease [60, 61]. Patients with asthma are more likely to develop lower respiratory tract symptoms after an upper respiratory tract infection. *Ex vivo*, bronchial epithelial cells from asthmatic airways are more susceptible to rhinovirus infection because of a deficient induction of the antiviral protein IFN-β [62]. A total of 147 people with asthma on inhaled corticosteroids with a history of virus-associated exacerbations, were randomized to 14-day treatment with inhaled IFN-β 1a (SNG001) or placebo within 24 hours of developing cold symptoms [63]. IFN-β treatment had no significant effect on asthma symptoms as primary endpoint, although it enhanced morning peak expiratory flow recovery, and reduced the need for additional treatment. Although the trial did not meet its primary endpoint, it suggests that inhaled IFN-β is a potential treatment for virus-induced deteriorations of asthma in difficult-to-treat patients [63].

## Conclusions

Asthma is a complex syndrome characterized by several phenotypic variants with different pathophysiological mechanisms and involvement of various cytokines. The new biological therapies have differentiated targets and mechanisms of action, so it should be mandatory to identify the different phenotypes and endotypes of asthma in the individual patients, in order to select the most appropriate pharmacologic approach in terms of long-term efficacy and safety.

To do this, we need help from the identification of biomarkers that can guide in choosing the best treatment, moving from a generalist approach to a more “tailored” therapy, particularly for severe asthma and other respiratory disorders refractory to usual care. Despite significant progress in the latest years, both in research and clinical application, however, the need for more effective treatments in neutrophilic and steroid-resistant asthma remains largely unmet.
